# Importance of QT/RR hysteresis correction in studies of drug-induced QTc interval changes

**DOI:** 10.1007/s10928-018-9587-8

**Published:** 2018-04-12

**Authors:** Marek Malik, Christine Garnett, Katerina Hnatkova, Lars Johannesen, Jose Vicente, Norman Stockbridge

**Affiliations:** 1 National Heart and Lung Institute, Imperial College, Dovehouse Street, London, SW3 6LY England, UK; 2 Division of Cardiovascular and Renal Products, Office of New Drugs, Center for Drug Evaluation and Research, US Food and Drug Administration, Silver Spring, MD USA; 3 Division of Clinical Pharmacology I, Office of Clinical Pharmacology, Center for Drug Evaluation and Research, US Food and Drug Administration, Silver Spring, MD USA

**Keywords:** QT/RR adaptation, QT/RR hysteresis, QTc interval, Heart rate correction, PK/PD modeling, Statistical simulation

## Abstract

QT/RR hysteresis and QT/RR adaptation are interlinked but separate physiological processes signifying how quickly and how much QT interval changes when heart rate changes, respectively. While QT interval duration is, as a rule, corrected for heart rate in terms of the QT/RR adaptation, the correction for QT/RR hysteresis is frequently omitted in studies of drug-induced QTc changes. This study used data from previously conducted thorough QT studies to investigate the extent of QTc errors caused by omitting the correction for QT/RR hysteresis, particularly in small clinical investigations. Statistical modeling approach was used to generate 11,000 simulated samples of 10-subject studies in which mixed effect PK/PD models were used to estimate drug-induced QTc changes at mean maximum plasma concentration of investigated compounds. Calculations of QTc intervals involving and omitting QT/RR hysteresis correction were compared. These comparisons showed that ignoring QT/RR hysteresis has two undesirable effects: (A) In the design of subject-specific heart rate corrections (needed in studies of drugs that change heart rate) omission of QT/RR hysteresis may lead to signals of QTc prolongation of more than 10 ms to be missed. (B) Irrespective of whether the investigated drug changes heart rate, omission of QT/RR hysteresis causes the widths of the confidence intervals of the PK/PD predicted QTc interval changes to be increased by 20–30% on average (exceeding 50% in some cases). This may lead to a failure of excluding meaningful QTc prolongation which would be excluded if using hysteresis correction. The study concludes that correction for QT/RR hysteresis should be incorporated into future studies of drug-induced QTc changes. Subject-specific heart rate corrections that omit hysteresis correction may lead to erroneously biased conclusions. Even when using universal (e.g. Fridericia) heart rate correction, hysteresis correction decreases the confidence intervals of QTc changes and thus helps avoiding false positive outcomes.

## Introduction

The principles and implications of the E14 regulatory guidance [1] have repeatedly been described and discussed [2, 3]. The concept of investigating drug-induced QTc interval changes by means of PK/PD modeling in early clinical studies [4] has obvious practical attraction [5] but, compared to the “standard” thorough QT (TQT) studies, it also brings challenges. Of these, one of the most obvious is the necessity of measuring the QTc values with high fidelity precision. This is because the regulatory review of the predicted QTc change at the mean maximum plasma concentration of the investigated drug needs to be based on the upper confidence interval of such a prediction. The width of the confidence intervals of the PK/PD predictions depends on both the number and the accuracy of independent observations. Since, compared to “standard” TQT studies, usual early clinical investigations involve fewer subjects per drug dose, it is impractical if not impossible to offset the effect of QTc inaccuracies by an increased number of subjects [2].

The accuracy of QTc data depends both on the accuracy of the initial electrocardiographic measurements and on the precision of heart rate correction. Different possibilities have been discussed for how to increase the accuracy of computerized measurements of the QT interval duration [6, 7]. Advanced strategies for heart rate correction are also known [8, 9]. Much lesser attention is paid to the measurement of RR values needed for heart rate correction. Most frequently, QT interval duration is corrected for the preceding RR interval or a small number of RR intervals (e.g., 3 cardiac cycles) are averaged [10].

This practice contradicts the physiologic knowledge that QT duration does not depend solely on the duration of the preceding RR interval or on a small number of preceding RR intervals [11, 12]. Thus, the dependency of QT interval on the underlying heart rate consists of two interlinked but separate processes; the QT/RR adaptation signifies how much QT interval changes when heart rate changes while the QT/RR hysteresis signifies how quickly QT interval changes when heart rate changes.

Different investigations of the QT/RR hysteresis proved that the QT interval duration is influenced by a long history of preceding heart rate. In healthy subjects, it takes at least 2 min for the QT interval duration to stabilize after an abrupt heart rate change [15].

This discrepancy between the overwhelming practice and the well-established physiological knowledge leads to the question of what is the magnitude of QTc errors caused by ignoring the QT/RR hysteresis and what are the implications of such errors for the accuracy of PK/PD models in clinical studies involving small number of subjects. Having this question in mind, we performed a statistical simulation study based on ECG measurements previously made in different TQT studies for which suitable data were available.

Different effects of QT/RR hysteresis omission on the accuracy of QTc data need to be considered. In investigations of drugs that change heart rate, subject-specific heart rate corrections are needed. If QT/RR hysteresis is omitted from the QT and RR data pairs that are used to construct the individual correction, the correction itself might be influenced. Independent of this, the omission of QT/RR hysteresis might influence the QTc data variability regardless of whether subject-specific or universal heart rate corrections are used.

## Methods

To address both these aspects, we divided the present investigation into two interlinked parts. Specifically, as further described in detail, we considered 4 different heart rate correction formulas to dealt with QT/RR adaptation and 4 different expressions of RR interval values to be used in the correction formulas to deal with QT/RR hysteresis. A schema of the investigation plan of the study is shown in Table 1.

**Table 1:** Investigative plan

	RR interval expression			
	Average of 3 RR intervals	10 s average of RR intervals	Universal QT/RR hysteresis	Subject-specific QT/RR hysteresis
Heart rate correction				
Fridericia	I and II	I and II	I and II	I and II
Linear individual	I	I	I	I
Log-linear individual	I	I	I	I
Curve-linear individual	I and II	I and II	I and II	I and II

Table shows the principal layout of the study. Four heart rate correction formulas were combined with four different ways of calculating the RR interval value that is used in the heart rate correction formula. This gives altogether 16 combinations of calculating QTc intervals. The study was divided into parts I and II and the table shows which combinations were investigated in which part of the study. See the text (and the Appendix) for the detailed definitions of the heart rate corrections and the RR interval expressions

### QT/RR hysteresis expressions

Different possibilities of quantifying QT/RR hysteresis have been published [12–14]. In both parts of this study, we used the exponential decay models [15] that calculate the RR interval duration used in the heart rate correction of a QT interval measurement as a weighted average of a series of RR intervals preceding the QT measurement. For the calculations, we used 5 min history of RR intervals preceding the QT measurement and considered two possibilities:

(A) Subject-specific QT/RR hysteresis in which the strength of the exponential decay model (and hence the weights of the preceding RR intervals) were optimized for each subject separately based on repeated QT and RR drug-free baseline measurements in the given subject [15, 16]. (See the Appendix for details.)
(B) Universal QT/RR hysteresis in which the same strength of the exponential decay model was applied to all subjects. As previously published [17], we used a universal model corresponding to the 95% adaptation of QT interval duration achieved in 2 min after an abrupt heart rate change. (See the Appendix for details.)
  To model the approaches of ignoring the QT/RR hysteresis, we also considered two further options:
(C) Correcting the QT interval measurements for the RR interval obtained as the average of RR intervals in a 10 s window preceding the QT measurement, and.
(D) Correcting the QT interval measurements for the average of 3 RR intervals preceding the QT measurement.

This means that for each QT interval measurement, we considered 4 different RR interval values with which to correct the QT duration.

### QT interval measurements

All QT interval measurements used in this investigation were obtained in previously conducted TQT studies. The measurements were made using previously described semi-automatic strategies [17] and involved both reconciliation of measurement discrepancies between different observers and morphological measurement adjustments to ensure that similar ECG waveforms were interpreted and measured systematically [18, 19]. For each QT interval measurement, a 5 min history of preceding RR intervals was also available allowing us to construct the RR interval expressions A to D.

### Heart rate corrections

In both parts of the study, different possibilities of calculating the QTc interval were used. Firstly, we considered both subject-specific heart rate corrections in the curve-linear form $QTc = QT + \frac{\delta }{\vartheta }(1 - RR^{\vartheta } )$ where coefficients $\delta$ and $\vartheta$ are optimized based on repeated drug-free baseline measurements in the given subject [9]. Secondly, we considered Fridericia correction $QTcF = QT/RR^{1/3}$ [20].

To complement the subject-specific heart rate corrections, we also used linear and log-linear formulas in the form $QTc = QT + \beta (1 - RR)$ and $QTc = QT/RR^{\alpha }$, respectively, where the coefficients $\alpha$ and $\beta$ are again optimized based on repeated drug-free baseline measurements in the given subject [21].

In the formulas of the subject-specific heart rate corrections, the coefficients $\alpha$, $\beta$, and $\delta$ represent the slope of the QT/RR relationship while the coefficient $\vartheta$ expresses the curvature of the QT/RR pattern.

This means that we have used one generic heart rate correction (the Fridericia formula) that is applied irrespective of the subject-specific QT/RR patterns and three subject-specific formulas derived from the subject-specific drug-free QT/RR pattern. Of these, the curve-linear formula incorporates the inter-subject differences in the curvatures of QT/RR patterns while the linear and log-linear formulas assume that shape (but not the slope) of the QT/RR relationship is the same in all subjects.

These different correction formulas were combined with 4 described possibilities of RR interval calculations (Table 1). Although all these calculations were made, not all the combinations make practical sense. In particular, as the subject-specific optimization of QT/RR hysteresis profile involves the computation of subject-specific heart rate correction, the combination of Fridericia correction with subject-specific hysteresis correction is of no practical value. On the contrary, the Fridericia correction can easily be combined with a universal hysteresis correction [17] as described in the Appendix.

### Study part I

As previously described [17], systematic ECG measurements in repeated day-time drug-free baseline 12-lead Holter recordings were obtained in 751 subjects (mean age 34 ± 10 years, 311 women). In each of these subjects, multiple QT interval measurements were made during each baseline recordings. All source studies that provided these data were approved by appropriate ethics bodies and all subjects gave written informed consent.

Using these data, we investigated how the different RR interval calculations A to D (involving and ignoring the QT/RR hysteresis) influence (a) the slopes of the subject-specific heart rate corrections and (b) the intra-subject standard deviations (SD) of the drug-free QTc and QTcF values. The slopes of the subject-specific corrections are important since steeper or shallower slopes predict larger or smaller QT interval changes associated with heart rate increases, respectively [8]. SD of drug-free baseline QTc values are important since larger spread of drug-free QTc data decreases the power of clinical investigations of drug-induced QTc changes [2].

### Study part II

For the purposes of this part of the study, drug-free baseline, on-placebo and on-treatment recordings were available from 11 clinical investigations of different drugs and drug doses. The investigations were all cross-over design and each was initially performed in 38–44 healthy subjects. In each subject, baseline, on-placebo and on-treatment 12-lead Holter recordings were available.

The 11 investigations were selected to represent a spectrum of different drug-induced heart rate accelerations. In each investigation, data of 15 time-points were available including the plasma concentrations of the investigated drugs. For each time-point and each subject, QT interval measurements (in drug-free baseline recordings and in recordings on placebo and on active treatment) were replicated 3–5 times. Heart rates at individual time-points were obtained from complete 5 min windows during which the study subjects were in supine resting positions. For each subject, complete drug-free baseline QT/RR profiles were also available to construct individual hysteresis and heart rate corrections. As with part I data, all source studies that provided the part II data were approved by appropriate ethics bodies and all subjects gave written informed consent. Since we only used the data to compare different hysteresis and heart rate corrections, the details of the investigations are of no relevance.

Different combinations of curve-linear, linear, and log-linear subject-specific and Fridericia corrections with different approaches to RR interval calculation were used to replicate the standard TQT evaluations. For these combinations, the baseline-corrected ∆∆QTc differences between placebo and active treatment were calculated together with 95% confidence intervals.

Consistent with the regulatory guidance [1], we calculated the time-wise matched difference between on-treatment QTc and corresponding baseline QTc (∆QTc_t_) and the time-wise matched difference between placebo QTc and corresponding baseline QTc (∆QTc_p_), and defined ∆∆QTc as ∆QTc_t_ − ∆QTc_p_.

The ∆∆QTc data of the different combinations of heart rate and hysteresis corrections were also used in a statistical simulation study that aimed at investigating the differences between the combinations in small clinical studies.

Specifically, from each of the source clinical studies, random groups of 10 subjects were selected. (Since both on-treatment and placebo recordings were available in each subject, this modeled “small” studies of cross-over design.) In each such randomly selected group, mean maximum plasma concentration of the investigated compound was obtained and a mixed effect linear PK/PD model [22] was used to estimate the ∆∆QTc and its upper one-sided 95% confidence interval at this plasma concentration. These ∆∆QTc estimates (at their upper 95% confidence intervals) and the widths of their 95% confidence intervals were compared between the different combinations of heart rate and hysteresis corrections. For each random group of 10 subjects, a mixed effect linear PK/PD model was also used to estimate the baseline-corrected heart rate change on active treatment (compared to placebo) at the mean maximum plasma concentration of the investigated compound [22]. The differences between the modeled ∆∆QTc estimates and between their confidence intervals were related to the modeled estimates of heart rate changes.

In each of the 11 source investigations, the random group of 10 distinct subjects was selected 1000 times (using Mersenne Twister pseudorandom number generator [23]) ensuring that the same random group was not selected repeatedly. This resulted in 11,000 individual simulation experiments. In each such random experiment, we evaluated the numerical differences between different ∆∆QTc estimates (i.e. estimates obtained with and without hysteresis correction) and the proportions of the widths of their confidence intervals. This made the assessment of the random experiments independent of the actual values of the plasma concentration (which naturally differed between different compounds).

### Statistics and data presentation

Numerical data are presented as mean ± SD. The majority of the results are presented graphically. Where appropriate, paired comparisons and correlations were evaluated using paired *t* test and Pearson correlation coefficient, respectively. Statistical results with p values ≤ 0.05 were considered statistically significant.

## Results

### Study part I

The total data of part I of the study involved altogether 897,570 individual QT interval and RR history measurements. On average, there were 1195 ± 293 measurements per subject. In 642 (85.5%) subjects, there were at least 1000 individual measurements. In each subject, the measurements were also sufficiently distributed across different heart rates and thus enabled studying individual QT/RR and hysteresis profiles.

**Fig. 1:**
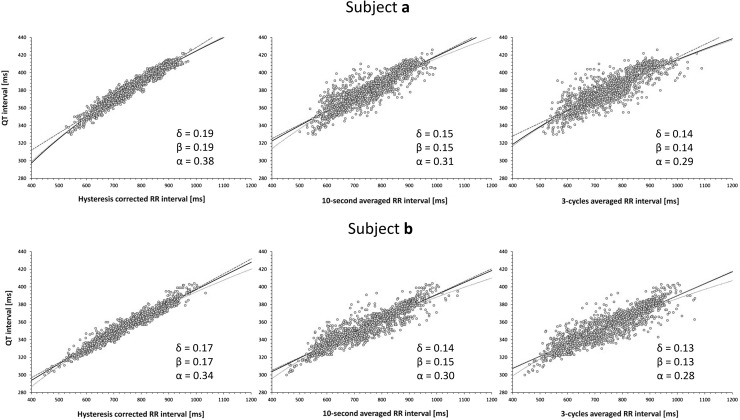
Examples of QT/RR relationships in two different healthy subjects. The scatter diagrams show QT/RR relationship of the very same QT interval measurements to RR intervals corrected for individually assessed QT/RR hysteresis (left panels), RR intervals obtained as the averages of cardiac cycles in the 10 s preceding the QT interval measurement (middle panels), and RR intervals obtained as the averages of 3 cardiac cycles preceding the QT interval measurement (right panels). In each panel, the bold blue line, dashed red line, and dotted green line correspond to the curve-linear, linear, and log-linear regressions, respectively. The slopes of the curve-linear (δ), linear (β) and log-linear (α) regressions are also shown in each panel. Note that the fit between QT intervals and RR intervals progressively worsens from left to right. Color illustration online

Figure 1 shows examples of how the omission of QT/RR hysteresis influences intra-subject QT/RR patterns and their regression modeling (which is the basis of subject-specific heart rate correction). As shown, the relationship of QT intervals to RR intervals corrected for QT/RR hysteresis is much tighter compared to the other RR interval expressions. Thus, omitting the QT/RR hysteresis from the assessment merely adds noise to the RR interval data. As with other cases of regression analysis, adding noise to the independent variable (i.e., RR intervals in the case of QT/RR regressions) makes the regression slopes shallower.

**Fig. 2:**
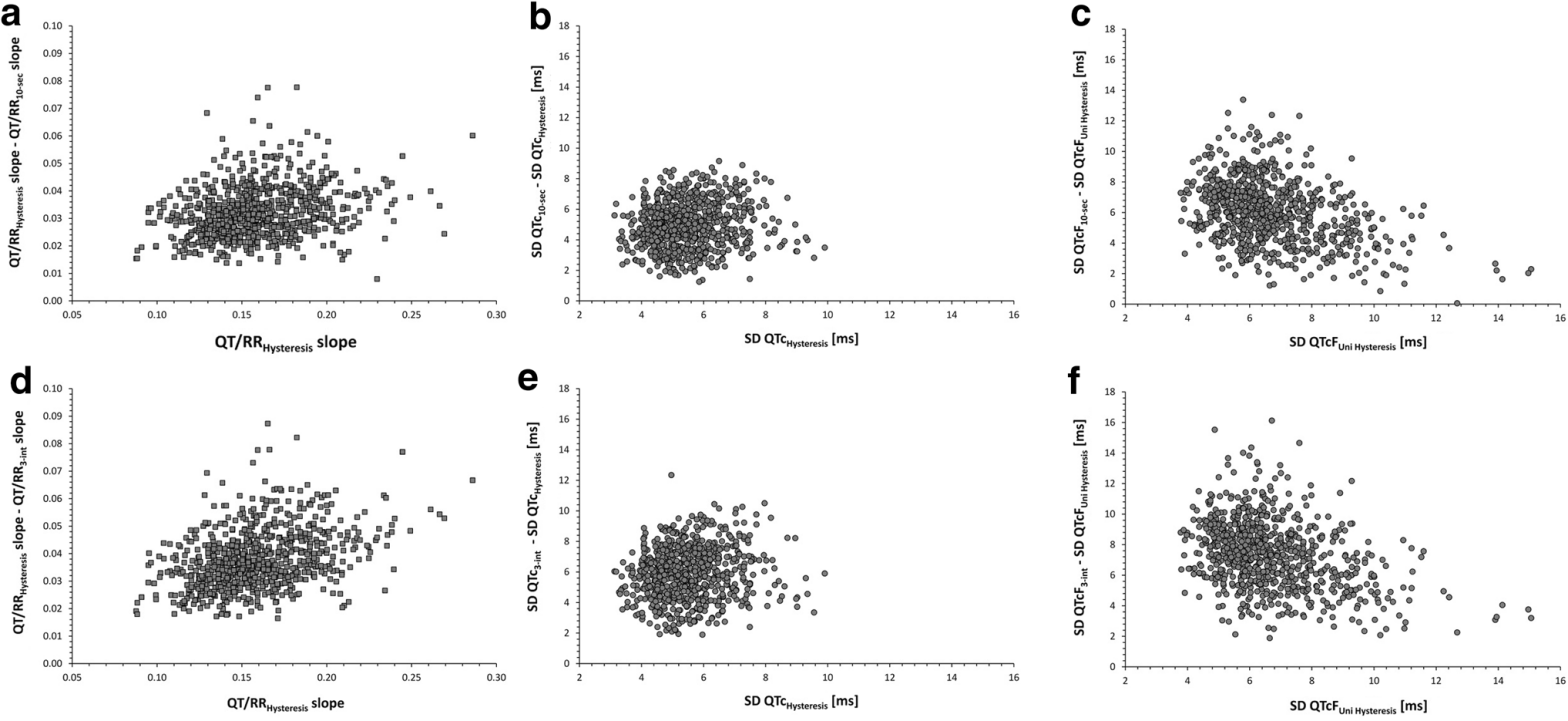
Summary of Part I investigation. Panels **a** and **b**: Decreases of intra-subject curve-linear QT/RR slopes caused by replacing QT/RR hysteresis correction with RR interval of preceding 10-second data (panel **a**) and RR intervals of preceding 3 cycles (panel **b**) shown against the curve-linear slopes involving subject-specific QT/RR hysteresis correction. Note that in all cases without exception, the slopes decreased. Panels **c** and **d**: Increases of intra-subject standard deviations of baseline drug-free QTc intervals corresponding to the slope comparisons shown in panels **a** and **b**, respectively. Note that in all cases without exception, the intra-subject standard deviation of QTc intervals increased when omitting the QT/RR hysteresis correction. Panels **e** and **f**: Increases of intra-subject standard deviations of QTcF intervals caused by replacing universal QT/RR hysteresis correction with RR interval of preceding 10 s data (panel **e**) and RR intervals of preceding 3 cycles (panel **f**) shown against the intra-subject standard deviations of QTcF intervals involving universal QT/RR hysteresis. QTc and QTcF - subject-specific curve-linear and Fridericia corrections, respectively; their subscripts describe the RR expressions used in the corrections. The corrections slopes in panels **a** and **b** are shown for RR and QT intervals in seconds. *SD* standard deviation

This has been confirmed by the analysis of regression slopes (i.e. the coefficients of subject-specific heart rate corrections Table 2, Fig. 2) and by the evaluation of the variability of QTc data (Table 3, Fig. 2). With all the investigated regression models, their slopes systematically and statistically significantly decreased from changing hysteresis corrected RR intervals for their 10 s or 3-cycles averages. Note in Fig. 2 (panels a and b) that the change towards lower slopes was systematic and that there was no subject in our dataset in whom the changes would be in the opposite direction. Note also that, for instance, with subject-specific linear heart rate correction, it is easy to calculate that coefficients $\beta$ of 0.161 and 0.122 (Table 2) lead to uncorrected QT interval decreases by approximately 40 ms and 30 ms if the heart rate accelerates from 60 to 80 beats per minute. (Differences of the same magnitude are also seen with coefficients $a$ of 0.35 and 0.26 in the subject-specific log-linear heart rate correction.) Thus, if the investigated drug leads to similar heart rate acceleration, drug-induced QTc interval prolongation of 10 ms can be missed if the shallower correction model is used.

**Table 2:** Numerical evaluations of QT/RR regression slopes in part I of the study

	RR interval expressions involved in QTc calculations				Intra-subject comparison with individual QT/RR hysteresis		
	Individual hysteresis	Universal hysteresis	10 s RR average	3-interval RR average	Universal hysteresis	10 s RR average	3-interval RR average
Curve-linear slope	0.159 ± 0.030	0.159 ± 0.030	0.127 ± 0.029	0.121 ± 0.028	0.000 ± 0.001	0.032 ± 0.009	0.038 ± 0.011
Linear slope	0.160 ± 0.030	0.161 ± 0.030	0.128 ± 0.029	0.122 ± 0.028	0.000 ± 0.001	0.032 ± 0.009	0.038 ± 0.011
Log-linear slope	0.347 ± 0.049	0.348 ± 0.050	0.276 ± 0.052	0.262 ± 0.051	0.001 ± 0.002	0.072 ± 0.022	0.085 ± 0.025

The table shows QT/RR regression slopes (i.e., the coefficients of subject-specific heart rate corrections) obtained when relating the drug-free baseline QT intervals to different expressions of RR intervals (left part of the table) and the decreases of these slopes when replacing the subject-specific hysteresis correction of RR intervals with other RR interval expressions (right part of the table). All the differences between subject-specific hysteresis corrections and RR expressions ignoring the QT/RR hysteresis (last two columns of the table) were statistically significant (p < 0.0001 for all). The slopes are for RR and QT intervals in seconds. The numerical values shown are population means ± standard deviations

**Table 3:** Numerical evaluations of QTc standard deviations in part I of the study

	RR interval expressions involved in QTc calculations				Intra-subject comparison with individual QT/RR hysteresis		
	Individual hysteresis	Universal hysteresis	10 s RR average	3-interval RR average	Universal hysteresis	10 s RR average	3-interval RR average
QTc SD [ms]	5.49 ± 1.10	5.52 ± 1.11	10.49 ± 1.94	11.41 ± 2.11	0.03 ± 0.11	5.00 ± 1.51	5.92 ± 1.68
QTc_L_ SD [ms]	5.67 ± 1.14	5.70 ± 1.15	10.68 ± 2.00	11.58 ± 2.17	0.02 ± 0.08	5.00 ± 1.52	5.91 ± 1.68
QTc_P_ SD [ms]	6.01 ± 1.21	6.04 ± 1.22	11.37 ± 2.07	12.31 ± 2.25	0.02 ± 0.01	5.37 ± 1.65	6.30 ± 1.81
QTcF SD [ms]	6.68 ± 1.71	6.72 ± 1.72	12.80 ± 2.09	14.08 ± 2.28	0.04 ± 0.09	6.08 ± 2.06	7.36 ± 2.25

The table shows intra-subject standard deviations of drug-free baseline QTc intervals obtained when correcting the QT intervals for different expressions of RR intervals (left part of the table) and the increases of these standard deviations when replacing the subject-specific hysteresis correction of RR intervals with other RR interval expressions (right part of the table)

All the differences between subject-specific hysteresis corrections and other RR expressions (last three columns of the table) were statistically significant (p < 0.0001 for all). The standard deviations are in milliseconds

The numerical values shown are population means ± standard deviations

*QTc* subject-specific heart rate correction based on the curve-linear correction model, *QTc*_*L*_ subject-specific heart rate correction based on the linear correction model, *QTc*_*P*_ subject-specific heart rate correction based on the log-linear correction model, *QTcF* Fridericia heart rate correction

As expected based on the observations made in Fig. 1, variability of drug-free QTc values also increases substantially and statistically significantly if hysteresis correction of RR interval values is omitted (Table 3, panels e and f of Fig. 2). Note in particular in Table 3 that in our data, the difference between SD of individually and hysteresis corrected QTc (SD of 5.5 ± 1.1 ms) and of Fridericia corrected QTcF that involved universal hysteresis correction (SD of 6.7 ± 1.7 ms) was much smaller than the difference between QTcF that involved and did not involve hysteresis correction (SD 12.8 ± 2.1 and 14.1 ± 2.3 ms for QTcF applied to RR expressions C and D, respectively). Note also in Fig. 2 (compare panels c and e, and similarly d and f) that the distribution of SD of QTcF with universal hysteresis correction was not only larger than SD of individually and hysteresis corrected QTc but also more widely distributed with cases of SD well above 10 ms (the largest value of SD of individually and hysteresis corrected QTc was 9.9 ms). These large SD cases were those where the curvature assumed by Fridericia correction was fitting the individual QT/RR distribution particularly poorly irrespective of which hysteresis correction was applied.

### Study part II

**Fig. 3:**
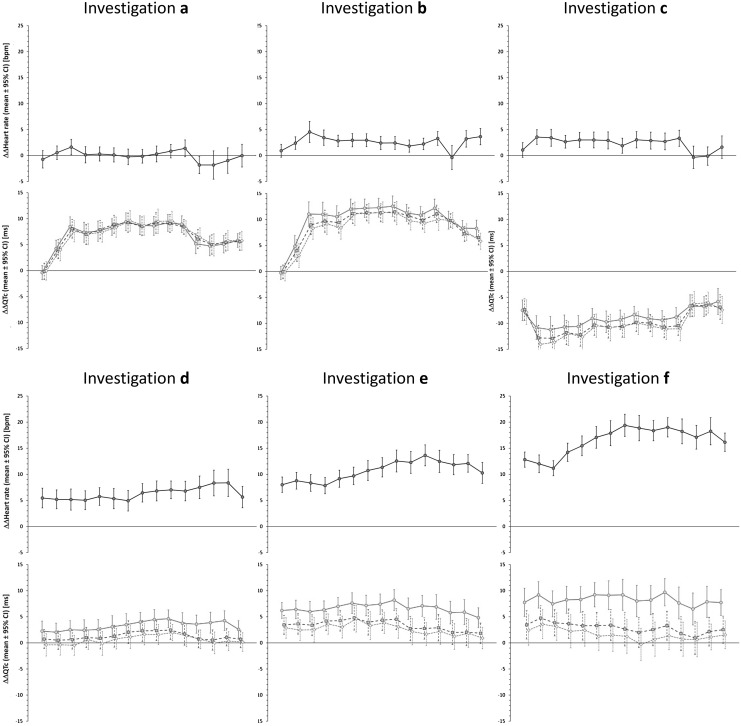
Representation of the scale of clinical investigations used in Part II of the study. For each of six clinical investigations the figure shows the ∆∆heart rate values (top panels), ∆∆QTc values based on subject-specific curve-linear heart rate corrections (bottom panels). In each panel, the ∆∆ values are shown (together with their 95% confidence intervals) for individual time-points of the source clinical study. In the bottom panels, the QTc corrections for RR intervals derived from subject-specific hysteresis profiles are shown with red circles and full lines, QTc corrections for RR intervals obtained from averages of preceding 10 s of cardiac cycles with blue squares and long-dashed lines, and QTc corrections for RR intervals obtained from averages of preceding 3 cardiac cycles with amber diamonds and short-dashed lines. Note that the larger the ∆∆heart rate value, the larger the discrepancy between ∆∆QTc values involving and ignoring the QT/RR hysteresis. Color illustration online

The 11 clinical investigations that we used in the part II of the study included a spectrum of different drug-induced heart rate changes - i.e., from no heart rate effects to about 20 beats per minute (bpm) increase in heart rate. A representative sequence of 6 of these investigations is shown in Fig. 3. The Figure allows comparing drug-induced heart rate changes and the discrepancies between study evaluations based on subject-specific heart rate correction including and ignoring the hysteresis correction of RR intervals. As predicted by the results of the Part I investigation, larger heart rate changes resulted in larger differences between the subject-specific heart rate corrections. The omission of QT/RR hysteresis led to systematically lower estimates of the drug-induced QTc change, irrespective of whether the drug was causing QTc prolongation or QTc shortening (compare study examples *b* and *c* in Fig. 3). Pooling of all the time-points of the studies together, the differences between ∆∆QTc point estimates based on study-specific heart rate corrections using and not using QT/RR hysteresis were significantly correlated with the point estimates of ∆∆heart rate (r = 0.98 and r = 0.97 for the differences between RR interval expressions A–C and A–D, respectively). Very similar differences between subject-specific heart rate corrections involving and not involving QT/RR hysteresis correction were also obtained with the subject-specific corrections based on linear and log-linear formulas (not shown in Fig. 3).

**Fig. 4:**
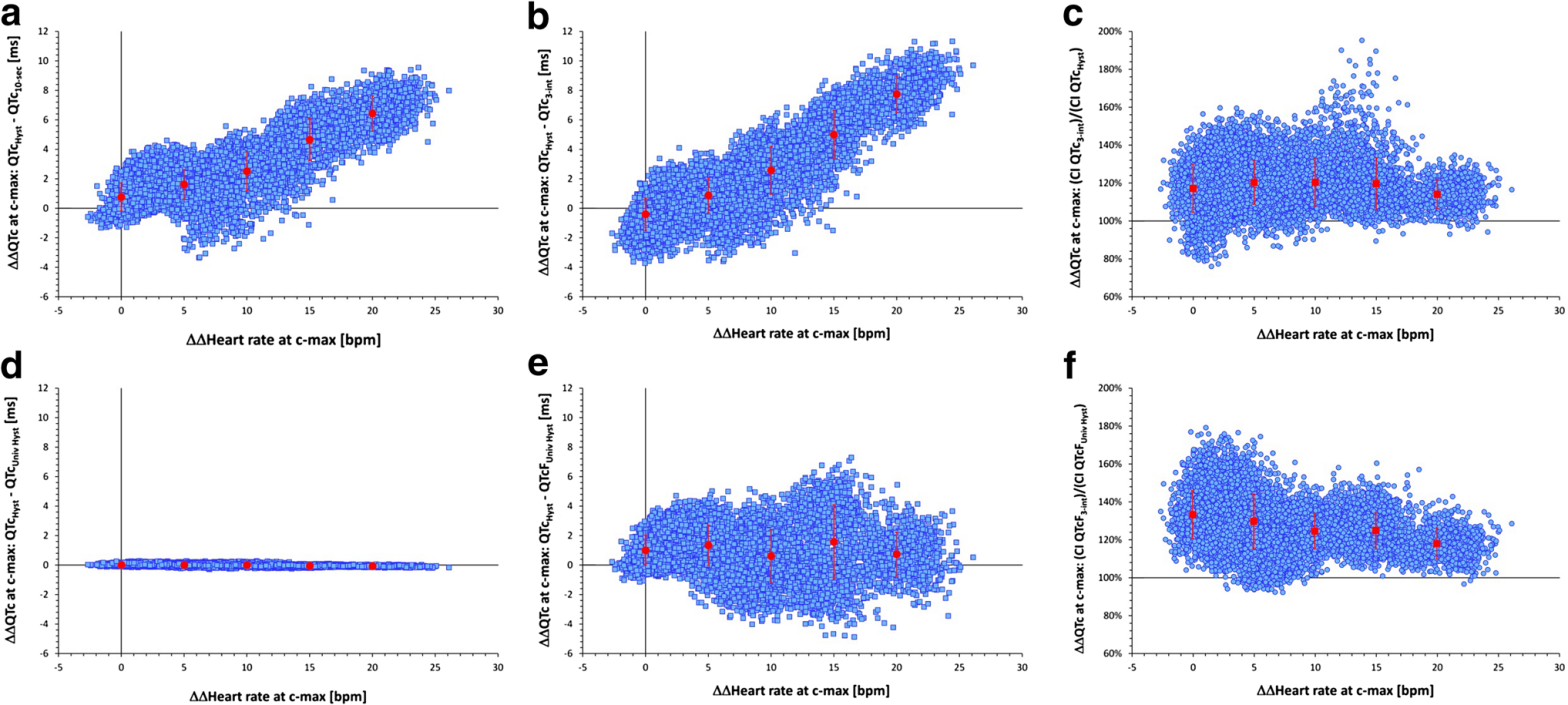
Results of statistical PK/PD statistical simulations of part II of the study. All the panels show scatter diagrams (in blue) investigating the dependency of modeled estimates of ∆∆heart rate (horizontal axes) at mean maximum plasma concentration (see the text for details). The vertical axes of Panels **a**, **b**, **c**, and** d** show the differences between the ∆∆QTc estimates based on subject-specific curve-linear heart rate corrections for RR intervals derived from subject-specific hysteresis profiles and: (panel** a**) ∆∆QTc estimates corrected for RR intervals obtained from averages of preceding 10 s of cardiac cycles, (panel** b**) ∆∆QTc estimates corrected for RR intervals obtained from averages of preceding 3 cardiac cycles, (panel** c**) ∆∆QTc estimates corrected for RR intervals obtained from universal hysteresis profiles, and (panel **d**) ∆∆QTcF estimates corrected for RR intervals obtained from universal hysteresis profiles. The vertical axes of Panels** e** and** f** show proportions between the widths of confidence intervals of ∆∆QTc estimates corrected for RR intervals derived from subject-specific hysteresis profiles and ∆∆QTc estimates corrected for RR obtained from averages of preceding 3 cardiac cycles (panel **e**) and ∆∆QTcF estimates corrected for RR intervals derived from universal hysteresis profiles and ∆∆QTcF estimates corrected for RR intervals obtained from averages of preceding 3 cardiac cycles (panel **f**). In each panel, the scatter diagrams are overlaid (in red) by mean ± standard deviations in bins of heart rate change of 0 ± 2.5, 5 ± 2.5, 10 ± 2.5, 15 ± 2.5, and 20 ± 2.5 beats per minute. QTc and QTcF - subject-specific curve-linear and Fridericia corrections, respectively; their subscripts describe the RR expressions used in the corrections. c-max – mean maximum plasma concentration of the compound studied in the source clinical investigation. Color illustration online

These observations were well reproduced in the statistical simulation study of the PK/PD models of heart rate and QTc changes at mean plasma concentrations. The results of the simulation experiments are shown in Fig. 4. The differences between the ∆∆QTc estimates including and ignoring QT/RR hysteresis were again highly correlated to the ∆∆heart rate estimates (r = 0.84 and r = 0.89 for panels a and b of Fig. 4, respectively).

The modeled point estimates of ∆∆QTc based on heart rate correction involving subject-specific QT/RR hysteresis and universal hysteresis (RR intervals expressions A and B) were minimal with differences of only 0.00 ± 0.07 ms which did not significantly differ from 0 (panel c of Fig. 4).

The statistical simulation study also allowed comparing the ∆∆QTc estimates based on subject-specific heart rate corrections and Fridericia corrections combined with universal hysteresis correction. These are shown in panel d of Fig. 4. The Figure shows that these differences were not related to the corresponding ∆∆heart rate estimates (r = − 0.03) and that while their averaged value over all the modeled experiments was not large, their spread increased at higher ∆∆heart rate reaching a spread between − 4.9 and + 7.3 ms. In more detail, in the bins of heart rate changes of 0 ± 2.5, 5 ± 2.5, 10 ± 2.5, 15 ± 2.5, and 20 ± 2.5 bpm, the mean, SD, and range of the differences between the ∆∆QTc estimates based on subject-specific heart rate corrections and Fridericia corrections combined with universal hysteresis correction were 1.0 ± 1.0 (− 1.1 to + 4.7), 1.3 ± 1.4 (− 3.5 to + 5.0), 0.6 ± 1.8 (− 4.0 to + 5.4), 1.6 ± 2.5 (− 4.9 to + 7.3), and 0.7 ± 1.5 (− 4.2 to + 6.1) ms, respectively. This is not surprising since, as previously discussed [17], the inaccuracy of Fridericia correction depends in each subject on the difference between drug-free the QT/RR curvature in the given subject and the QT/RR curvature expected by the Fridericia formula. Hence, the differences are driven by both the combination of the subject-specific QT/RR curvatures in study subjects as well as by drug-induced heart rate changes. Such combinations of subject-specific QT/RR curvatures were naturally dissimilar in different randomly generated groups of 10 subjects in part II of our study. We have therefore observed both positive and negative differences with their spreads increasing as the heart rate changes increased.

Finally, the modeling study also showed that the width of confidence intervals of ∆∆QTc estimates increased when ignoring the QT/RR hysteresis. The width of the confidence estimates of ∆∆QTc and ∆∆QTcF estimates increased by 19.0 ± 12.2 and 28.1 ± 12.8%, respectively (p < 0.0001 vs no increase for both) when changing the RR interval expressions from A to D for subject-specific heart rate correction (panel e of Fig. 4) and from B to D for Fridericia correction (panel f of Fig. 4).

## Discussion

The results of this investigation show that correction for QT/RR hysteresis should be incorporated into future investigations of drug-induced QTc interval changes. When subject-specific heart rate corrections are used to deal with drug-induced heart rate changes [8], omission of QT/RR hysteresis may have profound effect on the accuracy of such corrections. As shown, this may cause a meaningful drug-induced QTc prolongation to be missed. Irrespective of whether universally applicable heart rate correction (such as the Fridericia formula) or subject-specific heart rate corrections are used, omission of QT/RR hysteresis increases the variability of the QTc data that, in turn, increases the confidence intervals of the estimates of drug-induced QTc changes. When this cannot be offset by larger number of investigated subjects [2], e.g. in the early clinical studies [5], false positive observations of drug-induced QTc changes can be made.

While the statistical modeling study part of our investigation showed the effects of QT/RR hysteresis omission within the PK/PD modeling methodology, the effects are not limited to the PK/PD models. Irrespective of whether the evaluation of drug-induced QTc changes is based on PK/PD modeling or on the intersection–union test [1], omission of QT/RR hysteresis may limit the efficacy of the investigation and/or result in misleading conclusions.

Of particular importance is our observation that the differences between subject-specific and universal hysteresis corrections are negligible in studies of drug-induced QTc changes. Hence, contrary to detailed physiologic studies [14, 15], studies of drugs that do not lead to important heart rate changes can safely combine universal rate correction (e.g. Fridericia formula) with universal hysteresis correction without any practical decrease of accuracy. Universal hysteresis correction also does not require any baseline drug-free QT/RR profiling of individual study subjects and thus can easily be applied to early clinical studies in which only on-treatment or on-placebo recordings are available. (Note that in investigations of drugs that do lead to important heart rate changes, inaccuracies can also be caused by the application of universal heart rate correction including the Fridericia formula. Such possible inaccuracies are avoided by subject-specific heart rate corrections [8]).

Whilst the computerized QT interval measurement [6, 24] might occasionally be problematic, systematic computerized detection of QRS complexes and thus reasonably accurate measurement of RR intervals preceding any QT interval measurement does not cause any notable problems even in recordings in which noise pollution influences the quality of QT measurements. Hence, obtaining the data needed for applying universal hysteresis correction (i.e., RR interval histories of QT interval measurements—see the Appendix) does not complicate the standard studies of drug-induced QTc changes in any noticeable way.

There are other possibilities of improving the quality of QTc data. These include reducing the recording noise, using large numbers of multiple QT and RR measurements at each study time-point [25], measuring QT interval duration in multiple ECG leads [24], selection of measured ECG samples based on their noise contents [26], etc. Nevertheless, unless the effects of QT/RR hysteresis are fully eliminated by keeping the RR intervals preceding the QT measurement strictly constant (e.g. in the fixed rate atrial pacing studies [8, 27]) all these methodological approaches can meaningfully be combined with the hysteresis correction.

In addition to the heart rate correction by universal or subject-specific correction formulas, other possibilities have been proposed to investigate the drug-induced changes in the relationship between QT intervals and underlying heart rate [8]. Some of these can easily be made consistent with the concept of hysteresis correction. For instance, the so-called bin method [28] which compares uncorrected QT interval durations occurring at the same heart rate at baseline and on treatment can easily be modified so that the comparison are made not between QT intervals immediately preceded by the same RR intervals but preceded by the same hysteresis corrected RR intervals [29]. With universal hysteresis correction, this would not complicate the method in any way.

Other methods that are strictly based on relating QT intervals to the immediately preceding or following RR intervals (e.g. the studies of the T-end-to-Q-onset interval [30]) obviously suffer seriously from the lack of hysteresis correction, perhaps to an even greater extent than the RR expression option D that we have considered. Our results also appear to contradict the principles of the so-called one-stage approach to PK/PD modeling of combined heart rate and QT changes in the dependency on drug plasma levels (see the relevant section in [8]). Since the QT/RR relationship is not driven by simultaneously measured QT and RR intervals, incorporating such data in a combined PK/PD model contradicts the principles of QT/RR hysteresis and might lead to the same problems as shown in Figs. 3 and 4.

It might be argued that hysteresis correction is not needed if the heart rate preceding the QT interval measurement is kept stable by strict conduct of the clinical investigation. Unfortunately, we have previously reported that the concept of stabilizing heart rate by keeping the study subjects in strict motionless positions fails to achieve its goal in the vast majority of clinical investigations [17]. Indeed, all the 11 investigations that we have used in part II of this study followed a protocol of undisturbed supine positions before and during QT assessment time-points. Still, the effects of the hysteresis omission were well visible in the statistical simulation study (see panels e and f of Fig. 4).

As far as we are aware, the effects of incorporating and omitting hysteresis correction in studies of drug-induced QTc changes have not been investigated before. There is thus little data in the literature with which our principal results can be compared. Nevertheless, our findings are in close agreement with the physiologic principles of QT/RR hysteresis that have been established already decades ago [11, 31]. The appearance of larger discrepancies between subject-specific and Fridericia corrections at larger drug-induced heart rate changes (see panel d of Fig. 4) also corresponds to previously made observations [8].

## Limitations

Several limitations of our investigation need to be considered. Both parts of the study included data of healthy subjects. It has previously been reported that QT/RR hysteresis differs in cardiac patients and that the profile of hysteresis predicts cardiac risk [32, 33]. We are unable to comment on whether our observations and, in particular, the applicability of universal hysteresis correction would also apply to studies conducted in clinically well-defined populations of patients rather than healthy subjects. Similarly, we cannot comment on whether studies conducted in elderly would equally benefit from hysteresis correction. The effect of advancing age on the QT/RR hysteresis is presently poorly understood [9]. The universal hysteresis correction was previously tested [17] in the same population as now used in part I of this study. Nevertheless, the time-constant of the universal hysteresis correction corresponds not only to the physiologic observations [11] but also the hysteresis correction proposed by others [34, 35]. The clinical investigations that we included in part II of the study did not include drugs changing the heart rate very abruptly (e.g., within 1–5 min after dosing). While some experience with subject-specific hysteresis correction on abrupt heart rate changes exists [36], we cannot relate our findings directly to such situations. We also assumed that the subject-specific hysteresis correction remains the same during the day, i.e. that it is not influenced by autonomic variations which have occasionally been proposed [37]. The small variability of QTc data shown in Table 3 suggest that this assumption is reasonable at a practical level but we are unable to provide a detailed physiologic justification. The QT/RR hysteresis models that we have used do not distinguish between long-term and short-term hysteresis which has also been proposed [14]. Nevertheless, again, the QTc variability data shown in Table 3 suggest that the effect of such improvements in hysteresis description would have been only minimal and likely without practical importance.

## Conclusions

Despite these limitations, the study permits us to conclude that correction for QT/RR hysteresis should be incorporated into future investigations of drug-induced QTc changes. Regulatory assessment of such studies benefits from hysteresis correction since combination of subject-specific rate correction with hysteresis omission may miss important QTc prolongation signals. Evaluations of clinical studies also benefit from hysteresis correction since it improves the quality of QTc data and thus makes even smaller clinical studies more effective.

More specifically, two recommendations can be based on these conclusions:

1. If subject-specific heart rate corrections are used to evaluate a study that observed drug-induced heart rate changes (e.g. to avoid inaccuracy shown in panel d of Fig. 4), hysteresis correction is mandatory because without it, erroneously biased conclusions can be made. This applies to all subject-specific heart rate corrections irrespective of their mathematical form.
2. If fixed (e.g. Fridericia) heart rate correction is used, universal QT/RR hysteresis correction (see the Appendix for details) decreases the width of confidence intervals of ∆∆QTc values and thus helps avoiding false positive study outcome.

## Appendix

Handling of the QT/RR hysteresis depends on the way in which the RR interval values are obtained so that they can be subsequently used in a heart rate correction formula.

To facilitate implementation of the individually optimized and of universal hysteresis correction described in the text, we repeat here the previous published instructions [17, 35]:

If a QT interval reading is preceded by RR interval sequence $\left\{ {RR_{i} } \right\}_{i = 0}^{N}$ ($RR_{0}$ closest to the QT measurement), where $L = \mathop \sum \nolimits_{i = 0}^{N} RR_{i} \cong 300$ s, the exponential-decay hysteresis correction suggests correcting the measured QT interval for $RR^{'} = \mathop \sum \nolimits_{i = 0}^{N} \omega_{i} RR_{i}$, where for each $j = 0, \ldots ,N$,

$$ \mathop \sum \limits_{i = 0}^{j} \omega_{i} = \frac{{1 - e^{{\frac{{ - \partial \mathop \sum \nolimits_{i = 0}^{j} RR_{i} }}{L}}} }}{{1 - e^{ - \partial } }} $$

Method **(A)** of individually optimized hysteresis correction is based on finding the coefficient $\partial$ for each study subject separately [15]. This is performed in combination with the optimization of the individual heart rate correction formula, e.g. in the form

$QTc = QT + \frac{\delta }{\vartheta }(1 - RR'^{\vartheta } )$ which, incorporating the hysteresis, leads to the form of $QTc = QT + \frac{\delta }{\vartheta }\left[ {1 - \left( {\mathop \sum \nolimits_{i = 0}^{N} \omega_{i} RR_{i} } \right)} \right]^{\vartheta }$, where $\omega_{i}$ coefficients are as above. Using drug-free baseline data preceded by variable heart rates, coefficients $\delta$, $\vartheta$, and $\partial$ are optimized in such a way that the standard deviation of all baseline drug-free QTc values is minimal of all the possible combinations of $\delta$, $\vartheta$, and $\partial$ [16].

Method (B) of the universal hysteresis correction applies the same coefficient $\partial$ of all study subjects. This method assumes that the 95% adaptation of the QT interval after heart rate change occurs in 120 s [17] and corresponds to the numerical value of $\partial$ = 7.4622.

Using the same notation, the method **(C)** defines the $RR^{\prime}$ such that $RR^{\prime} = \frac{1}{m + 1}\mathop \sum \nolimits_{i = 0}^{m} RR_{i}$, where $\mathop \sum \nolimits_{i = 0}^{m} RR_{i} \le 10s$ and $\mathop \sum \nolimits_{i = 0}^{m + 1} RR_{i} > 10s.$

Method (D) uses a simple average $RR^{\prime} = \frac{1}{3}\left( {RR_{0} + RR_{1} + RR_{2} } \right).$
